# Thailand’s net-zero emissions by 2050: analysis of economy-wide impacts

**DOI:** 10.1007/s11625-023-01319-y

**Published:** 2023-04-28

**Authors:** Salony Rajbhandari, Pornphimol Winyuchakrit, Bijay Bahadur Pradhan, Achiraya Chaichaloempreecha, Piti Pita, Bundit Limmeechokchai

**Affiliations:** Thammasat University Research Unit in Sustainable Energy and Built Environment, Sirindhorn International Institute of Technology, Thammasat University, 99 Moo 18, Km. 41 on Paholyothin Highway, Khlong Luang, Pathum Thani, 12120 Thailand

**Keywords:** Asia-Pacific Integrated Model (AIM), Computable General Equilibrium model, Greenhouse gas emissions, Net-zero emissions, Thailand

## Abstract

This paper aims at exploring the economy-wide impacts of achieving net-zero greenhouse gas (GHG) emissions by 2050 in Thailand. This study developed a recursive dynamic Asia-Pacific Integrated Model/Computable General Equilibrium (AIM/CGE) model of Thailand for the assessment. The macroeconomic impacts of Thailand’s net-zero GHG emission targets by 2050 are analyzed relative to its 2-degree pathway. Results indicate that Thailand should put more effort in GHG mitigation actions to achieve the emissions peak by 2025 and net-zero GHG emissions by 2050. Improvement in energy efficiency; increasing electrification; expanding renewable energy utilization; deploying green hydrogen; bioenergy; carbon capture, utilization, and storage (CCUS); and behavioral changes are the key identified pillars of decarbonization to drive Thailand towards the pathways of net-zero emissions by 2050. Results show that there is a possibility of attaining net-zero GHG emissions by 2050 at the expense of an economic loss for Thailand. The gross domestic product (GDP) loss would be as high as 8.5% in 2050 to attain net-zero emissions. Lower productivity from the energy intensive industries such as petroleum refineries, coal and lignite mining, manufacturing industries, and transport are the key contributing sectors to the GDP losses. The price of carbon mitigation would shoot up to reach USD 734 per tCO_2_eq in 2050 from USD 14 per tCO_2_eq in 2025 to attain net-zero emissions in 2050.

## Introduction

Climate change is inevitable and is an issue of global concern. The impacts of climate change are happening, and they extend well beyond an increase in temperature, affecting ecosystems, human health, economic and social systems, and all regions around the world. Greenhouse gas (GHG) mitigation has become an increasingly important environmental issue for developing countries. Energy, being the vital commodity for economic development and the main factor for the rising anthropogenic GHG emissions, is closely interrelated with climate change. The Intergovernmental Panel on Climate Change (IPCC) indicates the urgency to reach net human-caused emissions of carbon dioxide (CO_2_) to reach net zero globally around 2050, including deep reductions in emissions of non-CO_2_ forcers, mainly methane (CH_4_), to limit the warming to 1.5 °C (IPCC 2022). Limiting warming to 1.5 °C requires rapid transformation of the energy system demanding a significant shift in the investment patterns.

Thailand, with a home to about 70 million people, is highly vulnerable to the impacts of climate change. The country has already been experiencing the adverse impacts of climate change through prolonged droughts, unprecedented storms, and floods along with the risks of sea level rise in the cities situated along the coastline. Having the second largest economy within the Association of Southeast Asian Nation (ASEAN) region, Thailand is the second largest energy consumer and is also the second largest in terms of the energy-related CO_2_ emissions (ECN 2016; ONEP 2018; Sandu et al. 2019; NESDC 2020). Over the last 4 decades, Thailand has scaled up the development ladder, uplifting itself from a low to an upper middle-income economy (World Bank 2020). As such, the country is rapidly growing and is most likely to bring about changes in the shape and structure of the energy consumption pattern and GHG emission curves in future.

Thailand has been taking concrete actions to avoid the impacts of climate change through the Nationally Appropriate Mitigation Action (NAMA) pledges, implementing a Nationally Determined Contribution (NDC) roadmap and building resilience in all sectors of the society. Thailand communicated the long-term low GHG emissions development strategy (LT-LEDS) to the United Nations Framework Convention on Climate Change (UNFCCC) in 2021 (MNRE 2021) and submitted the revised LT-LEDS in November 2022 (MNRE 2022). It outlines the key mitigation actions with enhanced mitigation efforts to achieve carbon neutrality by 2050 and net-zero GHG emissions by 2065. In order to meet the carbon neutrality by 2050 and net-zero emission goals, Thailand updated its NDC by increasing the GHG emissions reduction target to 40% in 2030 from the previous target of 25% (ONEP 2022).

Achieving net-zero emissions is a challenging task for Thailand with its energy system being mostly dominated by oil and natural gas. The country is heavily dependent on energy imports with almost 60% of its primary energy requirement met by imports (DEDE 2021). Natural gas-based power generation occupied a share of about 64% in the total power generation mix of the country, which is already a cleaner alternative as compared to other types of fossil fuels. By considering such cleaner mitigation actions early, Thailand is left with fewer available choices and is faced with higher marginal costs of reducing GHG emissions in the energy sector. In addition, limited information exists on the CO_2_ storage potential in Thailand. There are a few studies reporting CO_2_ storage capacity through carbon capture and storage (CCS) technologies of 10.3 giga tonnes of CO_2_ (GtCO_2_) in the country (ADB 2013; ERIA 2021). The natural forests of Thailand, which are the potential source for carbon sequestration, account for a total land area of 31.96% of the country equivalent to 16.4 million hectares. The net carbon sequestration from land use, land use change, and forestry (LULUCF) sector was reported to be 91.1 MtCO_2_ in 2016 (ONEP 2020).

There exist few studies on the potential of GHG emission reduction and pathways towards net-zero emissions in Thailand (Rajbhandari et al. 2019; Rajbhandari and Limmeechokchai 2021; Bahadur Pradhan et al. 2022; Chaichaloempreecha et al. 2022; Limmeechokchai et al. 2023). The focus of these studies varies in terms of analyzing the macroeconomic effects of limiting the GHG emissions in the pathways to achieve 2 and 1.5 °C targets (Rajbhandari et al. 2019); exploring the possibilities of achieving the carbon neutrality of the energy system (Rajbhandari and Limmeechokchai 2021); analyzing the technological implications and exploring the energy transition required to achieve net-zero emissions by 2050 (Bahadur Pradhan et al. 2022); assessing the impacts of carbon price and CCS on energy and GHG emissions of Thailand in the pursuit of achieving the 2 °C target (Chaichaloempreecha et al. 2022); and analyzing the energy and macroeconomic impacts of GHG emission reduction beyond the NDC targets (Limmeechokchai et al. 2023). However, the potential of GHG mitigation technologies and their economy-wide impacts of achieving net-zero GHG emissions in Thailand have not been sufficiently evaluated.

A transition towards net-zero economy is a necessary challenge for Thailand. This study makes an effort to develop Thailand’s net-zero GHG emission 2050 pathways based on the country’s aspiration to contribute to the global efforts and be in line with the Paris Agreement. Such deep decarbonization targets are likely to increase the adoption of several cleaner mitigation options and relevant policies that are expected to lessen the risk of energy burden and help to mitigate GHG emissions. However, the introduction of such options and policies would bring changes in the existing national economy in terms of the structure of production sectors, national welfare, carbon intensity, carbon price and other economic impacts. Thus, this paper attempts to analyze the economy-wide consequences of achieving net-zero GHG emissions in 2050 in the case of Thailand, one of the emerging economies in Asia. Identifying the strength and limitations of the possible decarbonization pathways to achieve net-zero GHG emissions by 2050 could provide useful insights to the policy makers.

## Methods

In this study, a multi-sector, recursive dynamic Asia-Pacific Integrated Model/Computable General Equilibrium model, named “AIM/CGE” model of Thailand, has been constructed to analyze the economy-wide implications of attaining net-zero GHG emissions by 2050. A soft linkage has been established between the bottom-up AIM/Enduse model and the top-down AIM/CGE model of Thailand to assess the macroeconomic aspects of technological innovation.

### The AIM/CGE model

The AIM/CGE model considered in this analysis uses the Mathematical Programming System for General Equilibrium Analysis (MPSGE) as the modeling language embedded within the General Algebraic Modeling System (GAMS) interface (Rutherford 1999). The AIM/CGE model is composed of a set of simultaneous equations without any objective functions. It uses mixed complementary problems for solution. The designed equations portray the behavior of various activities and sectors within an economy. The behavior of different sectors is captured using fixed coefficients. The behaviors of the production and consumption activities are captured by non-linear, first-order optimality conditions whose decisions are driven by the maximization of profits and utility, respectively. In addition, the formulated equations also consider a set of constraints that needs to be satisfied by the system as a whole and are basically known as the macroeconomic balance and the balance of payment (Fujimori et al. 2012). The AIM/CGE model has been widely adopted to analyze the economic and environmental implications of various energy and climate policies at the global, national, and sub-national levels (Wang et al. 2015; Wu et al. 2016; Dai and Masui 2017; Dong et al. 2017; Dai et al. 2018; Boonpanya and Masui 2020, 2021a, b; Malahayati and Masui 2021).

**Fig. 1:**
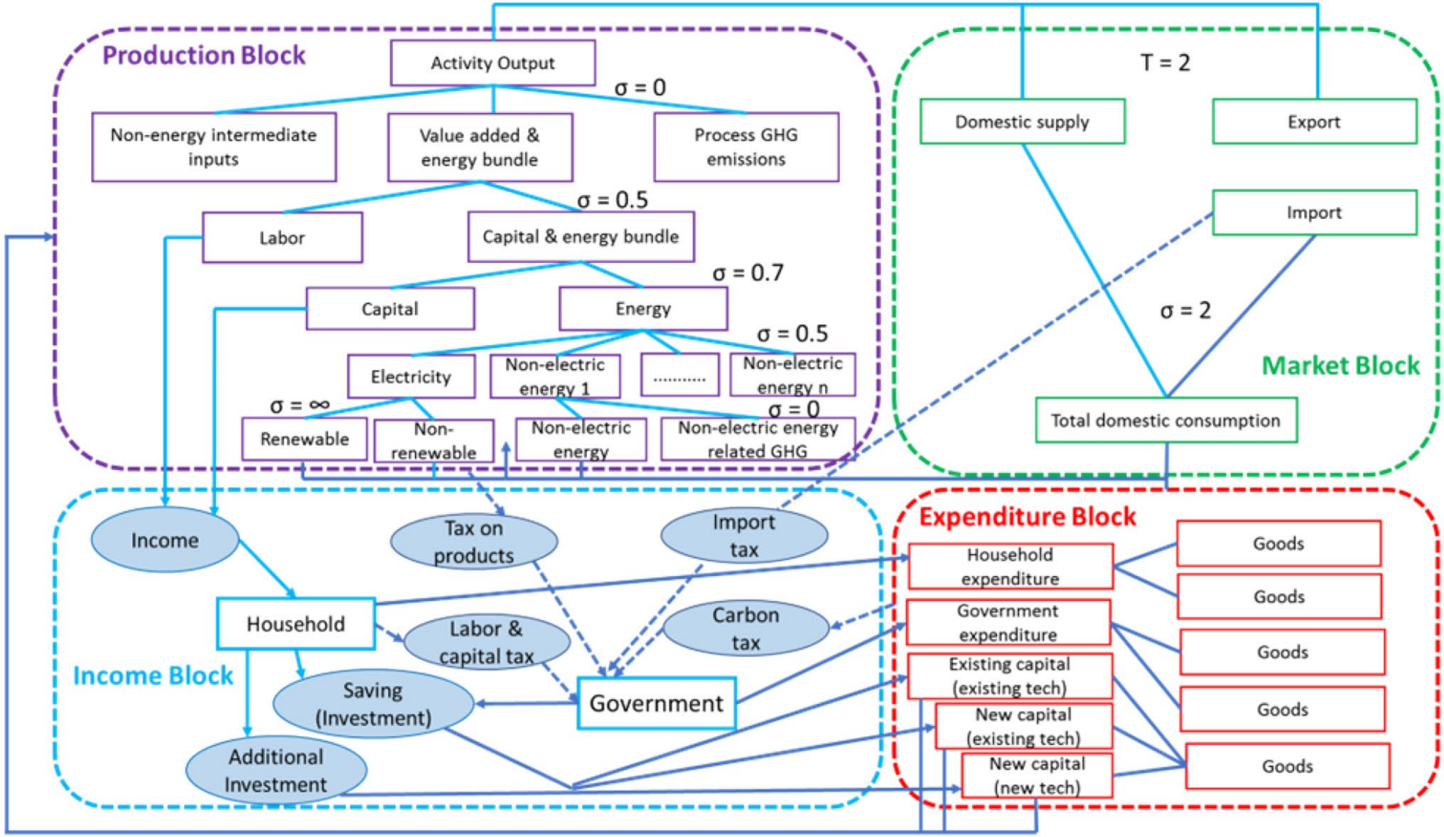
Structure of Thailand’s AIM/CGE model

The AIM/CGE model includes the following blocks: production, government and household income, and expenditure blocks, and a market block considering both domestic and international transactions (see Fig. 1). The activities of each production sector are represented by the nested constant elasticity of substitution (CES) function. The households maximize utility by consuming various levels of energy and non-energy goods, subject to the constraints of budget and commodity prices. The nested structure of household consumption for energy goods composite and non-energy goods composite are represented by the CES and Cobb–Douglas production functions, respectively. The government is entitled to collect taxes, including the direct taxes on household income, indirect taxes on gross domestic output, import tariffs on imports and other taxes. The government expenditure includes the government consumption, revenue transfers to public services and export rebates. The market block shows that the goods supplied to the domestic market can be either produced domestically or imported. The total domestic consumption in this study is modeled as a CES function of domestic goods supplied and imported goods. In case of exports, a constant elasticity of transformation (CET) function is considered to allocate total production of goods between exports and domestic demand for goods (see Fig. 1).

A soft linkage between the AIM/Enduse model and the AIM/CGE model has been established using the sector-specific GHG emissions and techno-specific data generated by the energy system model as an input to the AIM/CGE model. The AIM/Enduse model is a technology-rich, bottom-up recursive dynamic energy system model suitable for long-term energy analysis and is capable of quantifying both the global and local pollutants. The technological selection in the model is based on an optimization framework that minimizes the total system cost subject to various constraints. Details about the AIM/Enduse model can be obtained from Hanaoka et al. (2015).

### Sectoral classification in the AIM/CGE model of Thailand

The AIM/CGE model of Thailand is constructed using the input–output (I/O) table of 2015 to calibrate the model. The I/O table is obtained from the Office of the National Economic and Social Development Council (NESDC) of Thailand (NESDC 2021). The I/O table developed by the NESDC is originally classified into 180 × 180 sectors. For simplicity and saving of the computing time, the I/O table considered in this analysis is disaggregated into 31 production sectors of which five are energy sectors (see Table 1). The agriculture and forestry category is comprised of four sectors, while the industry, transport, service, and other categories include twelve, five, four, and one sectors, respectively.

**Table 1:** Sectoral classification in Thailand’s AIM/CGE model

**Non-energy sectors**	
Agriculture and forestry	Crops, livestock, forestry, and fishery
Transport	Railways, road transport, water transport, and air transport
	Other transport services
Service	Water supply system, communication, and trade
	Other services
Industries	Metal and non-metal ore, non-metallic products, basic metal, fabricated metal products, and machinery
	Food, beverages, and tobacco products; textile, paper, and printing; and chemical, rubber and plastic products
	Construction
	Other manufacturing products
Others	Other sectors
**Energy sectors**	
Coal and lignite, crude oil, petroleum products, gas, and electricity (including renewable and non-renewable)	

### Input data and assumptions

The development of the AIM/CGE model of Thailand requires other parameters besides the I/O data such as the energy balance, socioeconomic data, energy and technology prices, and emission factors. These parameters are exogenously provided to the AIM/CGE models. Both the import and export prices are also exogenously entered into the AIM/CGE models. It should be noted that Thailand’s CGE model constructed in this study adopts the “law of one price principle” for energy prices among the various sectors. The inconsistencies found in the energy consumption data across sectors between the I/O data and energy balance tables are reconciled to match the energy information provided in the energy balance of Thailand in 2015.

Thailand’s projected population during 2015–2040 is taken from the national statistics which assume declining fertility rates. According to the projections, the population is expected to gradually increase until 2030 and then decline afterwards (NESDC 2019). In this study, the population growth beyond 2040 is assumed to follow a similar declining trend. Based on the assumptions, the population of Thailand is estimated to reach 66.6 million in 2050 from 68.1 million in 2015, declining at a compound annual growth rate of 0.06% during the period of 2015–2050. During 2015–2019, the statistics show a decline in the percentage of working age population (15–59 years) from 65.7 to 64.4% (NSO 2020). To account for the future labor factor growth, the working age population in this study is assumed to follow the trend of declining population growth. The gross domestic product (GDP) projections considered in Thailand’s AIM/CGE model are based on the estimated average GDP growth rates of the “Power Development Plan 2018” and “Energy Efficiency Plan 2018” of Thailand (MOE 2020a, b). The projected GDP in this study is estimated to rise at an average growth rate of 2.71% during 2015–2050. Thailand’s economy saw a major downfall due to the Coronavirus pandemic in 2020, thereby shrinking the country’s GDP by 6.1%, making it the lowest expansion rate over the past 10 years. However, eventually, the economic growth is expected to gradually increase in this study.

The three GHGs, namely carbon dioxide (CO_2_), methane (CH_4_), and nitrous oxide (N_2_O), are treated as GHG emissions in the model. The I/O data and the estimated CO_2_, CH_4_, and N_2_O emissions in 2015 are used for estimating the GHG emission coefficients in 2015. The estimated emissions in 2015 are obtained from the national statistics (ONEP 2020). To avoid the problem of double counting, the GHG emissions from energy use and material use of fossil fuels are treated independently in the model. In the absence of country-specific elasticity values, the elasticities of substitution and transformation considered in the AIM/CGE model of Thailand are based on several international studies (Böhringer and Rutherford 1997; Paltsev et al. 2005; Benavente and Miguel 2016; Dai and Masui 2017; Li et al. 2017).

The capital is categorized into the existing stock and new investments in Thailand’s AIM/CGE model. The capital stock is updated by the model using the investments (fixed capital formation), depreciation, and economic growth. The study assumes 5% depreciation rate for the existing capital and 10% rate for the household’s energy equipment. The installed capital is assumed to be immobile and non-transferable to other sectors, whereas new investments can occur in any sector. The model assumes a linear relationship between the capital stock and the capital endowment. Labor is assumed to be fully mobile across the sectors within the economy. The model assumes fixed technological coefficients, no constraints on resources and efficient employment of all local resources. The model considers two different sets of technology options for each sector in each formulated scenario: existing technology with an energy productivity of 10% and efficient technologies with varying energy productivities of 20–70%. The non-energy input productivities of the various efficient technologies were considered to vary from 5 to 15%. The energy productivities in this study were estimated based on the cost-effectiveness of technology selection under the various scenarios in the AIM/Enduse model of Thailand, while the non-energy input productivities were assumed based on the effective technology options considered for GHG mitigation in the non-energy sector in the revised LT-LEDS study of Thailand (MNRE 2022).

### Scenarios

#### Reference scenario

The reference scenario (referred to as REF scenario from now onwards) considers the GHG emissions in Thailand’s 2-degree pathways as reported in the “Mid-century, Long-term Low Greenhouse Gas Emission Development Strategy (LT-LEDS)” of Thailand (MNRE 2021). As the Thai government is planning to revise the country’s energy plan considering the 2-degree strategy, this study has considered the GHG emissions of Thailand’s 2-degree pathway as a reference. The 2-degree scenario is already used as a business-as-usual scenario in the revised long-term strategy of Thailand submitted to the UNFCCC in November 2022. The impacts of Thailand’s revised LT-LEDS, specifically the 2050 carbon neutrality and the 2065 net-zero GHG emission targets, are analyzed relative to the 2-degree pathway (MNRE 2022).

Total GHG emission excluding the LULUCF is estimated to reach 390 million tonnes of carbon dioxide equivalent (MtCO_2_eq) in 2030 and 340 MtCO_2_eq in 2050 in the REF scenario. The net GHG emissions in this scenario are estimated to reach 220 MtCO_2_eq in 2050, with the LULUCF sector contributing emissions’ removal of 120 MtCO_2_. It should be noted that the sequestrations from the LULUCF sector are not determined endogenously in this study. This study assumes the carbon sequestration of 120 MtCO_2_ from the LULUCF sector during 2037 to 2050, as reported in the LT-LEDS studies of Thailand. The projected carbon sequestration is derived based on the National Strategy (2018–2037) of Thailand which aims to maintain the forest and green areas up to 55% of the country’s total land area (MNRE 2021, 2022).

#### Net-zero emission 2050 scenario

**Fig. 2:**
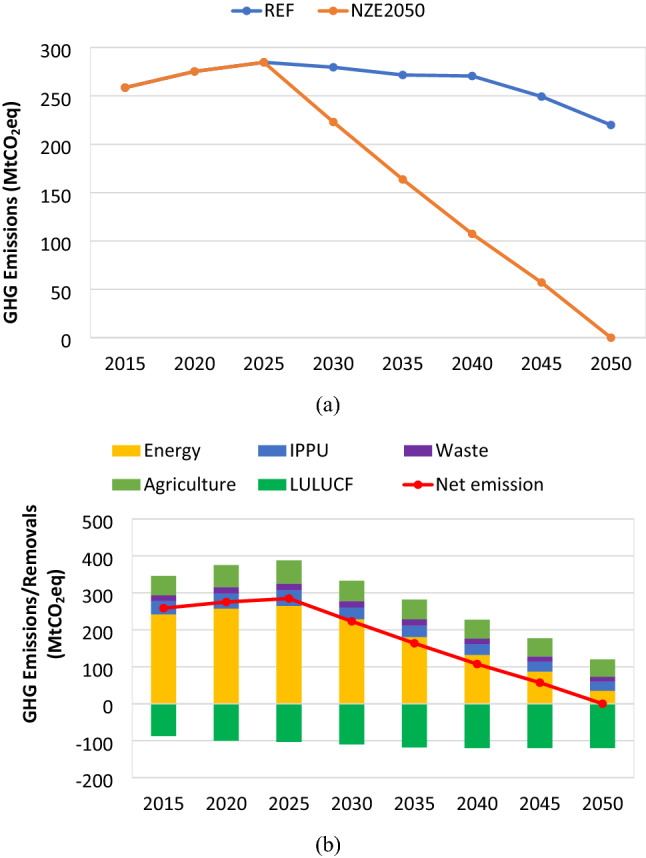
GHG emissions in REF and NZE2050 scenarios: **a** net GHG emissions, **b** GHG emissions/removals by sectors in the NZE2050 scenario

To achieve net-zero GHG emission in 2050, the LULUCF sector is expected to sequester 120 MtCO_2_ from 2037 to 2050 in the net-zero emission 2050 (NZE2050) scenario. As mentioned above, the capacity of carbon sequestration from the LULUCF sector is derived based on the forest and green area targets as mentioned in the National Strategy (2018–2037) of Thailand (MNRE 2022). The net GHG emissions are expected to reach the peak level of 285 MtCO_2_eq by 2025 in this scenario. Figure 2a presents the net GHG emissions profile in the REF and the NZE2050 scenarios during 2015–2050. If the REF emission pathways, which describes the 2-degree pathways, are extended beyond 2050, then it will reach net-zero emissions by 2090, as mentioned in the LT-LEDS of Thailand submitted to the UNFCCC in 2021 (MNRE 2021). As already mentioned above, in the revised LT-LEDS, Thailand has adopted the 2-degree pathways as a reference baseline which assumes reaching net-zero GHG emissions in 2090.

Figure 2b presents the sectoral GHG emissions and removals during 2015 to 2050 in the NZE2050 scenario. In the NZE2050 scenario, the total GHG emissions from sources would reduce to 120 MtCO_2_eq in 2050 from 346 MtCO_2_eq in 2015 and 375 MtCO_2_eq in 2020. The energy sector needs to undergo a major reduction of 86% in 2050 from its 2020 value. The industrial processes and product use (IPPU), waste, and agriculture sectors would need to cut their GHG emissions by 40, 26, and 21%, respectively, in 2050 in the NZE2050 scenario when compared to 2020. The energy sector would play a key role in GHG mitigation from 2025 onwards. The GHG emissions are expected to achieve a balance between GHG emissions by sources and removals by sinks in 2050. The NZE2050 scenario in this study assumes the introduction of both fossil fuel with CCS and bioenergy with carbon capture and storage (BECCS) technologies from 2040 onwards. Several energy-efficient and emerging technologies are considered in both the energy and non-energy sectors during the study period (i.e., 2020–2050) to help achieve the net-zero emissions in 2050.

## Results

### Effects on macroeconomics and welfare indicators

#### Impacts on GDP

**Fig. 3:**
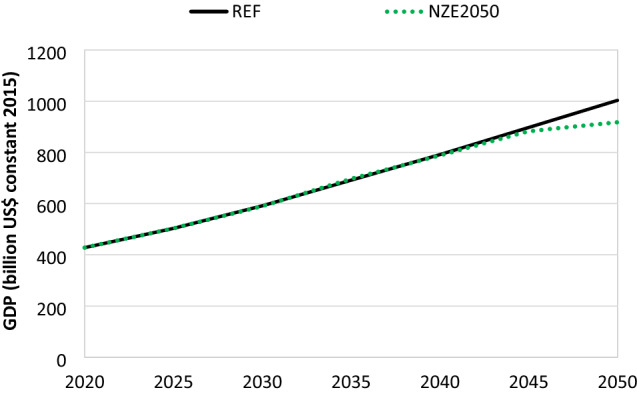
GDP in REF and NZE2050 scenarios

The GDP of Thailand would undergo an increase by more than twofold during 2020 to 2050 in the REF scenario, i.e., from 428 billion US$ in 2020 to 1003 billion US$ in 2050 (see Fig. 3). The total consumption including both the household and government expenditure demand would constitute more than 65% of the GDP in the REF scenario during 2020–2050. The net trade balance would remain negative during 2020–2050, portraying that Thailand would remain an export-oriented economy throughout the study period under the REF scenario. If in case, the economy of Thailand follows the business-as-usual path and not the 2-degree pathway, i.e., the REF scenario considered in this study, then to achieve the 2-degree pathway, the country would have to overcome the increasing GDP losses of 0.35% in 2025 to 6.72% in 2050. It should be noted that the business-as-usual path here refers to the continuation of the present emission trend without considering any climate mitigation policies. Since the Thai government is planning to revise the energy plan to consider the 2-degree strategy as a baseline, the results of net-zero 2050 scenario are compared to the 2-degree as a reference scenario in this study.

Results show that there is a possibility of attaining the net-zero GHG emissions by 2050 at the expense of an economic loss for Thailand (see Fig. 3). The economic losses tend to increase with the level of increased GHG mitigation in the NZE2050 scenario. Thailand’s economy would be able to reach the peak emissions in 2025 without any economic losses and would see a GDP gain of 0.8% in 2035 in the NZE2050 scenario. While in 2030, 2040, and 2045, the GDP losses would be 0.3, 0.4, and 1.6%, respectively. However, by 2050, the GDP loss would be as high as 8.5% in the NZE2050 scenario. However, in cumulative terms, the GDP loss during 2020–2050 would be 1.1% in the NZE2050 scenario. The cumulative economic loss in the NZE2050 scenario is attributed with introduction of the CCS technologies in 2040 and the cost-effectiveness of energy-efficient and emerging technologies.

**Fig. 4:**
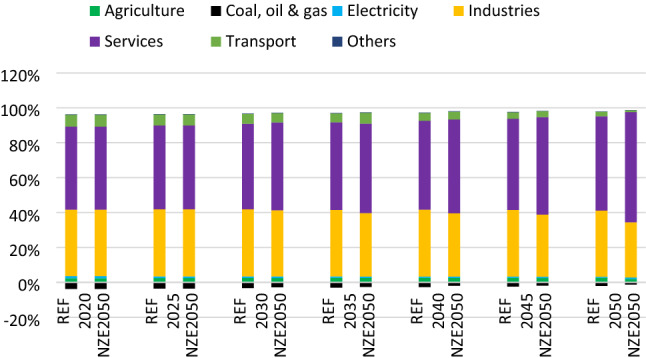
Sectoral share in total GDP in the REF and NZE2050 scenarios

The increases in productivity would lead to higher production output from the power, industries, and service sectors which would generate higher GDP, mostly around 2035 in the NZE2050 scenario. The increased productivity would cause an increase in the household consumption of goods and services in 2035 in the NZE2050 scenario when compared to the REF scenario. Figure 4 shows the sectoral share in total GDP across the REF and NZE2050 scenarios. The industries and service sectors occupy the major share in the total national GDP of Thailand in both REF and NZE2050 scenarios. Both the sectors accounted for about 93–96% share during 2020 to 2050, respectively, in the REF scenario. By 2050, both the sectors would account for more than 97% share in the NZE2050 scenario. Besides these sectors, the agriculture and forestry, transport, and electricity sectors are the other major sectors in the total national GDP of Thailand.

#### Impacts on household and government consumption

**Fig. 5:**
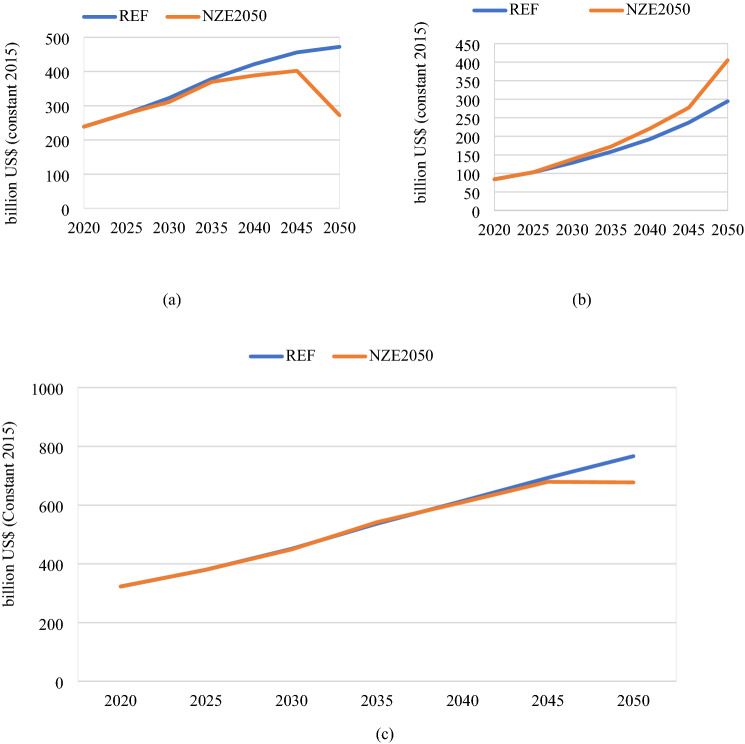
Impacts on consumption in the REF and NZE2050 scenarios: **a** household consumption, **b** government consumption, and **c** total consumption

Both household and government consumption would undergo an increase during the study period. The household final consumption expenditure representing consumer spending would increase from 239 billion US$ in 2020 to 472 billion US$ in 2050, i.e., an increase of twofold in the REF scenario (see Fig. 5a). The GHG mitigation pathways following the NZE2050 scenario would lead to a major decline in household consumption towards 2050. The household consumption would decline by 3.6% in 2030 to 42.4% in 2050 in the NZE2050 scenario. During 2020–2050, the cumulative drop in household consumption would be 8.2% in the NZE2050 scenario when compared to the REF scenario. This study showed that as the level of GHG emission reduction increases towards achieving net-zero emissions in 2050, the consumer spending on goods and services declines due to economy downturns. The major decline in the household consumption of goods and services occurs in the industries, transport, and service sectors during 2045–2050. Among the industries, the major decline of consumer spending occurs in the non-metallic products; construction; chemical, rubber, and plastic products; fabricated metal products; and food, beverage, and tobacco industries.

The government consumption would increase by more than threefold during 2020–2050 in the REF scenario, i.e., from 84 billion US$ in 2020 to 295 billion US$ in 2050 (see Fig. 5b). Government consumption needs to increase significantly to attain the NZE2050 scenario. The government consumption would undergo an increase of more than fivefold in the NZE2050 scenario by 2050. In cumulative terms, the government consumption would be needed to increase by 13.1% in the NZE2050 scenario during 2020–2050. It should be noted that the government usually increases spending when the economy is in recession or downturn to boost economic activities and regulate the economy.

The total consumption, including both the household and government consumption, would undergo more than twofold increase in the REF scenario, i.e., from 323 billion US$ in 2020 to 767 billion US$ in 2050 (see Fig. 5c). In cumulative terms, the total consumption during 2020–2050 is estimated to be declined by 1.5% in the NZE2050 scenario. Results show that the total consumption is found to have declined by 11.7% in 2050 in the NZE2050 scenario. The decline in the household consumption pattern of goods and services occurring in the industries and the transport sector is the major cause behind the fall in the total consumption around 2050.

#### The welfare loss

The drastic decline in the household consumption would lead to a sharp increase in the welfare losses in the NZE2050 scenario (see Table 2). The cumulative welfare loss would reach 6.8% in the NZE2050 scenario during 2020–2050. With the occurrence of a steep decline in the GHG emissions during 2030–2050, the welfare losses are likely to be higher during this period. The steeper GHG emission reductions towards 2050 would cause a larger welfare loss of about 34.8% in 2050. This is because the sharp decline in the GHG emissions towards 2050 would cause a drop in household consumption by 3.6% in 2030 to 42.4% in 2050 due to steep decline in the consumer spending for goods and services. The model results show that technological innovations should be incorporated with demand reductions to achieve the net-zero GHG emissions targets in 2050.

**Table 2:** Welfare loss in the NZE2050 scenario compared to the REF scenario

% Loss in cumulative terms		
2020–2050	2025–2050	2030–2050
6.8	7.6	8.9

% Loss in		
2030	2040	2050
3.1	7.2	34.8

### Economic implications of net-zero emission 2050 pathways

#### Price of carbon emissions

**Fig. 6:**
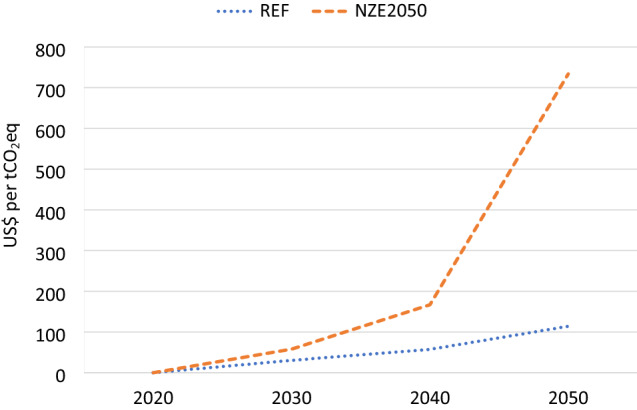
Variations in carbon price across the REF and NZE2050 scenarios

The prices of carbon or the GHG mitigation costs presented in Fig. 6 reflect the stringency of mitigation requirements to achieve the NZE2050 pathway of Thailand. The price of carbon would vary across the REF and NZE2050 scenarios. The level of technology introduction and selections lead to a wide variation in the prices of carbon in both the scenarios. Higher carbon prices are required to attain the NZE2050 scenario during 2025–2050. The carbon price would shoot up to reach US$ 734 per tCO_2_eq towards 2050 to attain net-zero emissions in the NZE2050 scenario.

The wide range of GHG mitigation prices between the REF and NZE2050 scenarios depends on many aspects, including GHG mitigation targets, availability of technology, characteristics of technology in terms of investment costs and rate of deployment (Rogelj et al. 2015; Riahi et al. 2017). A higher GHG price implies larger reductions in the household consumption of goods and services. Such large reductions of household consumption could be minimized by switching towards cleaner and more efficient energy resources and technologies. A study found that the prices of GHG mitigation are sensitive to the limited availability of technologies and vary according to the non-availability of BECCS technologies (Bauer et al. 2020). The deployment of technologies and mitigation strategies are also sensitive to the varying discount rates (Rogelj et al. 2018). The result of this analysis is based on a 10% discount rate for energy equipment and a 5% depreciation rate for the existing capital. The socioeconomic conditions and policy assumptions affect the price of GHG mitigation.

Delaying the climate policies will hurt economic growth. If the right GHG mitigation measures are implemented without delay and higher polluting technologies such as coal-fired power plants are phased out on time, then the economic costs could be small. However, if the transitions to widespread deployment of renewables are delayed, then the costs can be much greater. Therefore, such delayed mitigation policies and measures may even result in a further increase in the prices of carbon. The increased carbon prices in such cases are mainly because of a need for stronger efforts to counterbalance the higher emissions. However, studies reveal that there is no unique path for the price of GHG mitigation and it varies across studies (Rogelj et al. 2018).

### Mitigation opportunities for achieving net-zero emissions by 2050

**Fig. 7:**
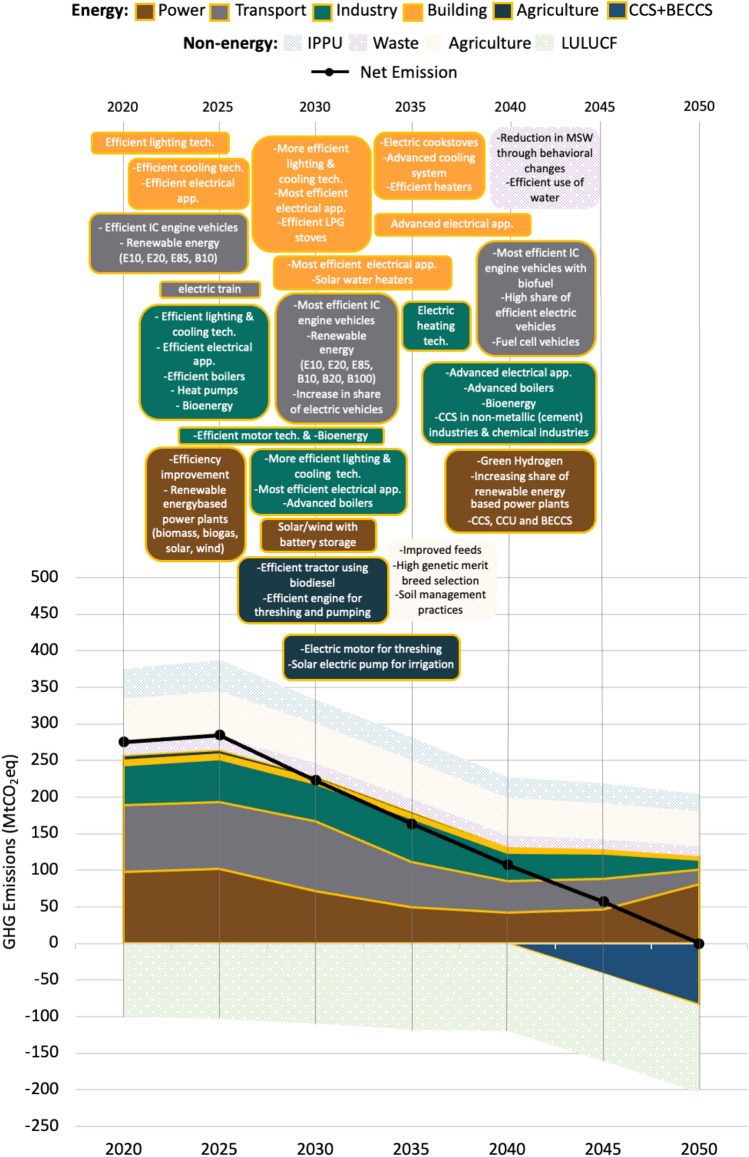
Key mitigation actions in the pathway to achieve net-zero emissions in 2050

The International Energy Agency (IEA) identified key pillars of decarbonization to achieve net-zero emissions which include energy efficiency; electrification; renewable energy development; hydrogen and hydrogen-based fuels; bioenergy; carbon capture, utilization, and storage (CCUS); and behavioral changes (IEA 2021). Figure 7 shows the timeline of mitigation actions that are needed for Thailand’s energy and non-energy sectors to achieve net-zero GHG emissions by 2050. The mitigation opportunities in the energy sector presented in this section are obtained from the bottom-up technology-rich AIM/Enduse energy system model of Thailand. It should be noted that the AIM/Enduse model is soft linked with the AIM/CGE model in this study using the sector-specific GHG emissions and techno-specific data generated by the AIM/Enduse model. The mitigation measures in the non-energy sector are based on the revised long-term strategy of Thailand submitted to the UNFCCC (MNRE 2022). The energy sector is a major component of Thailand’s transition towards net-zero GHG emissions by 2050.

Though renewable energy-based technologies such as solar photovoltaics (PV), wind, biomass cogeneration plants, and biogas are already in use, their shares in total power generation are still low at present. The share of renewable energy-based electricity generation is estimated to be 67% of total electricity generation in 2050 to achieve net-zero GHG emissions in 2050. Coal phase-out and negative emission technologies would be necessary in Thailand to achieve the NZE2050 pathway. The fossil- and biomass-based power plants equipped with CCS technologies would be needed from 2040 onwards to achieve the NZE2050 scenario. The CCS technologies would play a vital role in mitigating emissions from the power generation sector by 2050. Together the CCS and the BECCS technologies would abate about 84 MtCO_2_eq by 2050 in the NZE2050 scenario. Together the coal- and natural gas-based power generation technologies equipped with CCS would generate about 76.8 terawatt-hour (TWh) of electricity in 2050, accounting for about 14% share in the total power generation mix of Thailand in the NZE2050 scenario. Similarly, the BECCS technology would contribute to 29.6 TWh of electricity generation in 2050, accounting for about 5% share in the total power generation mix in the NZE2050 scenario. Additional mitigation technologies such as the solar PV with battery storage, fuel cell power plants, and green hydrogen technologies would be necessary in the power sector for achieving net-zero GHG emissions by 2050.

This study identified energy-efficiency measures, fuel-switching, and electrification of end-use technologies as the decarbonization measures in the industrial sector. The electrification of end-use technologies in this sector includes replacing the non-electricity using technologies with electricity-based technologies. Fossil fuel-based heating applications are partially or completely replaced by bioenergy and electricity in the manufacturing industries. The CCS technologies in the cement and chemical industries will play a significant role in decarbonization to achieve net-zero emissions by 2050.

The decarbonization opportunities in the building sectors lie in the improvement of energy efficiency of end-use technologies. Energy-efficiency improvement of lighting, cooling such as air-conditioners and refrigeration and cooking technologies, and electrical appliances would play a key role in decarbonization. Electrification of end-use cooking occurs through a shift from liquified petroleum gas (LPG)-based cooking to electric cooking. Solar water heating technologies also emerge as a promising option for decarbonization from 2030 onwards. Diesel, gasoline, and electricity are the major fuels used for energy purposes in the agricultural sector. Improvement in energy efficiency, electrification of end-use devices for threshing, pumping, motors and tractors, and solar water pumping systems are the three major GHG mitigation options in the agricultural sector.

Gasoline, diesel, compressed natural gas (CNG), LPG, and fuel oil are the major fuels used in the transport sector in Thailand. As the government has imposed mandatory blending of biofuels with gasoline and diesel in Thailand, gasohol blends (E10, E20, and E85) and biodiesel blends (B7 and B20) are commercially in use in the country at present (Khanunthong 2021; USDA 2022). Cleaner and efficient technologies such as hybrid, plug-in hybrid, electric, and fuel cell vehicles are the decarbonization opportunities in the transport sector. In addition, efficiency improvement of the internal combustion engine (ICE) vehicles following EURO5 and EURO6 standards and promoting use of liquid biofuels are other cleaner options which provide emission reduction benefits in the transport sector. However, major challenges lie in electrifying the transport sector. The electrification of the transport sector would first require decarbonization of the power sector. The electrification of the transport sector without increasing the share of cleaner and renewable energy-based electricity generation might lead to negligible GHG emissions reduction or even higher GHG emissions (MNRE 2022).

Reduction in municipal solid waste (MSW) through behavioral changes, community solid waste management, efficient use of water, waste to energy, and industrial wastewater management are the identified measures for GHG mitigation in the waste sector. The key prioritized mitigation measures in the agriculture sector includes improved rice cultivation practice, improved feeds for ruminant animals, high genetic merit breed selection, soil management practices, and lifestyle changes (MNRE 2022).

## Discussion

This paper was drawn with the aim of analyzing the economy-wide impacts of achieving net-zero emissions in 2050 in Thailand. This study developed and used the dynamic AIM/CGE model as a tool to examine the macroeconomic impacts of achieving net-zero emissions in 2050. The introduction of GHG countermeasures considered in this study provides benefits in terms of GHG emission reductions; however, the imposition of such strategies would be economically inefficient because of GDP and welfare reductions.

A study by Kainuma et al. (2017) suggested that the earliest possible attainment of the global GHG emissions peak would be necessary to achieve the long-term temperature goal in order to reduce the intensity of mitigation efforts that would be required due to a delayed peak. This analysis showed that to attain net-zero GHG emissions by 2050, Thailand should attain the emissions peak by 2025 that would be compatible with the stringent target of achieving net-zero emissions by 2050 in the pursuit of meeting the Paris Agreement goals. The revised LT-LEDS of Thailand aims to achieve net-zero GHG emissions by 2065. According to the revised LT-LEDS, the GHG emissions from sources are expected to reach the peak level of 388 MtCO_2_eq by 2025, with the energy sector playing the key role in mitigating the GHG emissions after 2025 (MNRE 2022). However, achieving emissions peak by 2025 will be a very challenging task for Thailand and requires emissions mapping to trace and track the status of GHG emissions to make climate mitigation action faster and easier.

Achieving net-zero emissions by 2050 requires huge leaps in cleaner energy innovation. Reaching net zero by 2050 requires further rapid deployment of available technologies as well as widespread use of technologies that are not available on the market yet. Together, the three technology areas: advanced batteries, hydrogen electrolyzers, and carbon capture and storage, would make a vital contribution in GHG emissions reduction during 2040–2050. In addition to the need of research and development for their wide-scale adoption, these technologies will also require construction of infrastructure at a larger scale. As such, government’s research and development spending needs to be prioritized as well as increased to rapidly accelerate the demonstration and deployment of cleaner energy innovation. The critical areas of prioritization include electrification, hydrogen, CCS/CCUS, and bioenergy with CCS (BECCS). The results highlight that the adoption of CCS technologies in both the fossil fuel-based and bioenergy-based power plants, along with the increment in other renewable energy-based power generation, using solar and wind, would provide significant potential to lower GHG emissions from power generation. However, high uncertainties and challenges remain in the wide adoption of CCS technologies in both the electricity generation and manufacturing industries.

However, there are several limitations in this study. The simulation results presented in this study are based on the input–output data in 2015 and under the underlying assumptions of fixed technological coefficients, constant return of scale, no constraints on resource availability, and efficient employment of all local resources. Results show that achieving net-zero GHG emissions in 2050 calls for a widespread deployment of BECCS. The expansion of the biomass plantation, however, not only demands a larger area of landmass but also requires technological improvements to efficiently use bioenergy resources. In addition, implementation of afforestation would be necessary on a large scale to sequester carbon emissions. This study does not provide limitations on the availability of land, energy, and water resources.

The contribution of the LULUCF sector to achieve net-zero emissions in 2050 is considered to be 120 MtCO_2._ This amount of carbon sequestration considered in this study is taken from the long-term strategy of Thailand and is not determined endogenously by the Thailand’s AIM/CGE model (MNRE 2021, 2022). This is another limitation of this study. If the contribution of the LULUCF sector as emissions removal is less than the considered target, then larger reduction from emissions’ sources would be required from other economic sectors to meet the net-zero target. This uncertainty may impact the socioeconomic conditions causing further distortions of GDP and other macroeconomic indicators. The uncertainty also lies in the growth of carbon stock in the forests which can be at a lower rate than expected. This suggests that forest utilization practices with a focus on maintaining the long-term carbon stock growth should be addressed. Furthermore, additional measures to minimize the risk associated with drought conditions and forest fires are needed to sustain the forestry sector growth to increase the carbon accumulation potential.

Maintaining clear communication between the government and the private sector involved in the cleaner energy business could stimulate the penetration of renewable energy and energy-efficient technologies and thereby encourage private sector investments. This could help in lowering the cost of GHG mitigation and macroeconomic impacts. In addition, the foreign investments and support in the form of finance, technology transfer, capacity building, raising awareness, and adaptation related to climate change could enhance the abilities to implement GHG mitigation measures at a much faster pace.

## Conclusions

This paper finds that making a transition to net-zero GHG emissions is a very challenging task for Thailand. Despite the current gap between the target and the reference scenario, this study shows that there are pathways of achieving net-zero emissions by 2050. Thailand’s pathway to net-zero emissions by 2050 as outlined in this paper requires the government to strengthen and then implement the energy and climate policies successfully. Results suggest that the pathway to net-zero emissions requires immediate and massive deployment of all the available and emerging clean and efficient energy technologies. Though the modeling exercise presented in this study shows the possibility of achieving net-zero emissions by 2050 through the deployment of already available and future possible technologies, uncertainties remain in the large-scale deployment of the efficient and future advanced technologies in the real world. The real-world implementation would pose significant challenges in terms of technological deployment and dissemination, and would call for huge supply-side as well as demand-side investments.

Both CCS and BECCS are identified to be promising technologies to decarbonize the energy system of Thailand. However, the commercial deployment of CCS technologies still remains a matter of uncertainty. Besides, viability and available storage capacity of CCS in Thailand are still uncertain. There is a need to assess the optimum utilization capacity of renewable resources such as solar and wind to further explore their roles in GHG mitigation analysis. Research on negative carbon technologies and emerging options such as CCS, BECCS, and green hydrogen are still inadequate in Thailand and need to be addressed before concluding that these are promising solutions to attain net-zero emissions in 2050.

Results show that a net-zero future is achievable by 2050 at the expense of GDP loss, requiring a drastic cut in household consumption accompanied by welfare losses in Thailand. Such a drastic cut would have severe impact on the country’s economy in the longer run giving rise to unemployment, declining real income, and reduced production of goods and services. Analysis shows that when the economy is in downturn, government should increase spending in welfare activities which could help in lowering the cost of GHG mitigation as well as the macroeconomic losses. However, some no-regret opportunities also exist that would not increase the GHG burden such as the improvement in energy efficiency of lighting and cooling technologies, increased usage of efficient electrical appliances and boilers, increased share of renewable energy-based power plants, increased share of efficient internal combustion engine, biofuel operated and electrical vehicles, usage of solar water heaters, and efficient LPG cookstoves. These technologies are found to be cost-effective in the modeling analysis from 2025 onwards even with a small or negligible policy push. Besides, changing energy demand behavior plays a prominent role in reducing the GHG burden and hence minimizes the macroeconomic losses. However, several challenges persist with demand-side management programs focusing on behavioral patterns of energy consumption.

Adequate and equitable financial, technological, and capacity-building support remain key drivers to drive Thailand towards a climate neutral economy. The transition towards net-zero emissions is a demanding task which requires a clear communication between the government and private sector involved in the clean energy business to stimulate the deployment of low or no carbon technologies, thereby attracting private sector investments. Finally, collaboration and partnership at the national, regional, and international levels are imperative to help Thailand to achieve the net-zero targets sustainably.

## Data Availability

The input-output table considered in this study is freely available in the original source (NESDC 2021). Population projections were obtained from (NESDC 2019), GDP growth rates were obtained from (MOE 2020a, b) and base year emissions were obtained from (ONEP 2020).
